# Study protocol: Evaluating the impact of a rural Australian primary health care service on rural health

**DOI:** 10.1186/1472-6963-11-52

**Published:** 2011-03-01

**Authors:** Rachel Tham, John S Humphreys, Leigh Kinsman, Penny Buykx, Adel Asaid, Kathy Tuohey

**Affiliations:** 1 Monash University School of Rural Health, PO Box 666, Bendigo Victoria 3552, Australia; 2 Elmore Primary Health Service, 46 - 48 Jeffrey Street, Elmore Victoria 3558, Australia

## Abstract

**Background:**

Rural communities throughout Australia are experiencing demographic ageing, increasing burden of chronic diseases, and de-population. Many are struggling to maintain viable health care services due to lack of infrastructure and workforce shortages. Hence, they face significant health disadvantages compared with urban regions. Primary health care yields the best health outcomes in situations characterised by limited resources. However, few rigorous longitudinal evaluations have been conducted to systematise them; assess their transferability; or assess sustainability amidst dynamic health policy environments. This paper describes the study protocol of a comprehensive longitudinal evaluation of a successful primary health care service in a small rural Australian community to assess its performance, sustainability, and responsiveness to changing community needs and health system requirements.

**Methods/Design:**

The evaluation framework aims to examine the health service over a six-year period in terms of: (a) Structural domains (health service performance; sustainability; and quality of care); (b) Process domains (health service utilisation and satisfaction); and (c) Outcome domains (health behaviours, health outcomes and community viability). Significant international research guided the development of unambiguous reliable indicators for each domain that can be routinely and unobtrusively collected. Data are to be collected and analysed for trends from a range of sources: audits, community surveys, interviews and focus group discussions.

**Discussion:**

This iterative evaluation framework and methodology aims to ensure the ongoing monitoring of service activity and health outcomes that allows researchers, providers and administrators to assess the extent to which health service objectives are met; the factors that helped or hindered achievements; what worked or did not work well and why; what aspects of the service could be improved and how; what benefits have been realised and for whom; the level of community satisfaction with the service; and the impact of a health service on community viability. While the need to reduce the rural-urban health service disparity in Australia is pressing, the evidence regarding how to move forward is inadequate. This comprehensive evaluation will add significant new knowledge regarding the characteristics associated with a sustainable rural primary health care service.

## Background

### The Australian rural health service context

Australia is a vast continent with a population of approximately 22 million people of which 3.5 million are spread across 1500 small rural and remote communities and 7.5 million square kilometres. Isolated rural communities throughout Australia are experiencing demographic ageing, an increasing burden of chronic diseases[1], and de-population as families move to larger cities. In addition, many of these rural communities are struggling to maintain viable and comprehensive health care services due to lack of service infrastructure, transport difficulties[2], and health worker shortages due to high levels of staff turnover and difficulties in recruiting new health workers[3]. In many cases rural communities are either foregoing care, travelling to larger regional centres or depending on irregular visiting services. As a consequence, Australia's rural and remote communities face significant health service disadvantages compared with metropolitan regions[4].

The vast distances separating small communities throughout rural Australia provide enormous challenges for authorities responsible for servicing population health needs as there are conflicts between ensuring operational efficiency and cost-minimisation, whilst maintaining effective and equitable delivery of accessible health services. Traditional urban health service models are proving to be unsustainable. Undoubtedly, there is no "one-size-fits-all" solution to meeting the diverse health needs of rural Australian residents and the range of service models needed is likely to vary between communities. Hence it is necessary to investigate models of health service delivery to ensure equitable access to care and reduce the health differential between rural and metropolitan people[5].

Research has shown that a primary health care approach yields the best health outcomes in situations characterised by limited resources[6]. As a result, the need for sustainable comprehensive primary health care, characterised by multi-disciplinary team approaches, is urgent in rural communities. In response, a wide variety of innovative health care models have evolved and been trialled in rural and remote areas[3]. However, whilst research has identified the requirements for sustainable rural and remote primary health care services[7], few rigorous longitudinal evaluations have been conducted to systematise them over time; assess their transferability to other regions[8]; assess how health services continually evolve to address ongoing changes in the external environment; monitor the effect of comprehensive primary health care on health service utilisation behaviour and health literacy of their communities[9]; or better understand how or when primary health care services can redress the poorer rural health status.

Given these gaps in knowledge it is vital to understand which rural health services 'work well, where and why' and are sustainable over the long-term in order to inform rural health service policy, assist with planning sustainable health services in other rural communities[10] and contribute to the equitable delivery of health care services that are likely to bring about improved health outcomes.

This paper describes the study protocol of a comprehensive longitudinal evaluation of a successful primary health care service located in a small rural Australian community to assess its performance, sustainability over time, responsiveness to changing community needs and health system requirements, and its impact on community health behaviours, health outcomes and community viability.

## Methods/Design

### Setting: The Elmore Primary Health Service (EPHS)

The EPHS is located at Elmore in central Victoria (Australia) 170 kilometres from the capital city, Melbourne. While the population in Elmore in 2006 was only 693[11], the EPHS provides care to nearly 3,000 patients from surrounding districts up to 125 kilometres from Elmore. The EPHS is a single-entry point, comprehensive primary health care model formed in 2001 by a partnership between the private medical practice and the local, publicly funded, community health services after the closure of the local hospital and loss of the local doctor in 1994. The model combines health care, community coordination and outreach services, is financed by public and private funding and is delivered by a multidisciplinary team of, amongst others, doctors, nurses, physiotherapists, psychologists and podiatrists[12]. This service has grown in contrast to the rural national trend of service closures[13] and can be an exemplar for other communities to emulate.

### Evaluation framework: domains, components and indicators

The longitudinal evaluation framework evolved from Donabedian's quality of care paradigm linking structure, process and outcome[14] and a conceptual framework for primary health care performance assessment developed originally by Sibthorpe[15], as reported in another publication[16]. This evaluation aims to examine the health service over a six year period in terms of:

a. Key **structural **domains:

• *Health service performance *characteristics;

• The *sustainability *of the health service organisation and function; and

• *Quality of care *that the service provides across the health promotion, treatment and rehabilitation spectrum.

b. Key **process **domain:

• The effect of the service on *health service utilisation *and *satisfaction*.

c. Key **outcome **domain:

• The effect of the service on *health behaviours, health outcomes *and *community viability*.

A key aspect of the evaluation was the identification and development of unambiguous sentinel indicators for each component of these key domains that can be reliably and validly operationalised so that data can be routinely collected and the health service monitored over time. The recent health systems reform process being undertaken in Australia has highlighted the difficulties in developing appropriate and valid accountability and performance benchmarks[17]. Despite potential indicators being easily identified on the conceptual level, it can be extremely difficult to operationalise them in forms for which data can be routinely and unobtrusively collected whilst being beneficial to health outcomes and health services. Hence our indicator selection was guided by significant health services research conducted by the Canadian Institute for Health Information[18]; the Australian National Health Performance Committee[19]; the Australian National Health and Hospital Reform Commission[17]; the Royal Australian College of General Practitioners[20]; the Australian Institute of Health and Welfare [21]; and Wakerman et al[22].

Drawing on this evidence, sentinel indicators for assessing the structural domains: health service performance (Table 1); sustainability (Table 2); and quality of care (Table 3), were developed in accord with the evaluation framework. As described in each table, each domain is divided into components, for which sentinel indicators were selected based on their technical merits and validity as identified in the research literature and their applicability in the health service setting[23] - that is, the data can be routinely collected, validated, and extracted reliably from the data sources identified for this study (health service record audits, community surveys, interviews and focus group discussions).

**Table 1:** Health Service Performance Domain

*Components*	*Reason for selection*	*Sentinel indicator items selected*	*Data source*	*References*
***Accessible***	The importance of geographical proximity to services; and timely and affordable access to routine and emergency care.	• Distance to nearest GP	Survey	AIHW [21]
		• Distance to usual GP	Survey	
		• Operating hours	Audit	Campbell S [26]
		• After hours services	Survey	CIHI [18]
		• Availability of emergency care	Survey/Audit	RACGP [20]
		• Availability of bulk-billing	Audit	AIHW [21]

***Appropriate***	To assess the comprehensiveness of services in dealing with a whole of health approach to health care.	• Availability of disease prevention, health promotion, early identification, sub-acute, acute and rehabilitation services.	Audit	AIHW [21] CIHI [18] NHHRC [17] RACGP [20]
		• Availability of female GPs	Audit	AIHW [21]

***Effective***	To assess extent to which a service's disease prevention interventions are achieving the desired results within an expected timeframe.	• Immunisation coverage	Audit	NHHRC [17]
		• Cervical cancer screening coverage	Audit	
		• Use of chronic disease registries for diabetes mellitus, asthma and hypertension for timely planning and disease management.	Audit	Campbell S [26]; CIHI [18]

***Responsive***	To assess the extent to which respectful care is provided that promotes dignity, privacy, safety and community empowerment.	• Service response to cultural and other specific needs of people utilising the service	Interviews	RACGP [20]
		• Community input into service planning	Interviews Focus group	NZRCGP [27], RACGP [20]

***Continuous***	The importance of providing uninterrupted, coordinated care across programs, providers, and organisations over time	• Choice of GP or nurse	Survey	RACGP [20]
		• Age-specific health assessments	Audit	CIHI [18]
		• 45 year old health check		
		• Use of integrated care plans for diabetes, asthma and depression	Audit	
		• Use of recall and reminder systems	Audit	RACGP [20]

***Efficient***	The importance of achieving desired results with the most cost-effective use of service resources.	• Electronic billing system	Audit	RACGP [20]
		• Electronic medical records	Audit	RACGP [20]

**Table 2:** Sustainability Domain

*Components*	*Reason for selection*	*Sentinel indicator items*	*Data source*	*References*
***Workforce***	The importance of having a workforce that is appropriate in number, volume and distribution and is responsive to emerging needs.	• Staff profile- Numbers and FTE. • Staff length of stay • Succession planning • CPD activities	Audit Interview Audit	AIHW [21] Wakerman[22] RACGP [20]

***Linkages***	Efficient and effective co-ordination between providers and between services is essential for continuity of care and service sustainability.	• Centralised electronic medical records • Care integrated with external agencies and mainstream programs	Audit/Interview	Wakerman et al [22]; CIHI[18];

***Infrastructure***	Infrastructure and ICT needs to be appropriate to the service, its catchment population and monitoring and reporting requirements.	• Uptake of Information and Communication Technology (ICT)	Audit	CIHI [18]; Wakerman et al [22]

***Funding***	Financing and provider remuneration should be appropriate, sustainable and clearly identified within program budgets to maximise service efficiencies and adequate to meet identified community health needs.	• Funding sources: public, private, other • Service providers' remuneration methods	Audit Audit	Wakerman et al [22] CIHI [18]

***Governance, management and leadership***	Good governance and leadership have been identified to be integral to service sustainability	• Governance structure and processes and a risk management plan in relation to service sustainability need to be clearly defined, implemented and reviewed.	Interview/documentation	RACGP [20], Wakerman et al [22]
		• Level of accreditation	Audit	NHPC [19]

**Table 3:** Quality of Care Domain

*Components*	*Reason for selection*	*Sentinel indicator items*	*Data source*	*References*
***Primary prevention***				
Cervical cancer screening	Cervical smear tests improve the early detection and treatment of cervical cancer and improve survival and quality of life.	Cervical cancer screening coverage	Audit	NHHRC [17]
Immunisation	Immunisation is a very important public health measure that can prevent the spread of common infectious diseases that cause significant morbidity and mortality.	Immunisation coverage:	Audit	AIHW [21], NHHRC [17]
		• Children		
		• Older adults (65+years)		
		• Aboriginal and Torres Strait Islanders		

***Secondary prevention***				
Recording of modifiable risk factors in medical records	Many common chronic diseases are preventable if the risk factors are identified and managed so that there can be improved health status and reduced health inequalities and need for health care.	• Smoking status	Audit	AIHW [21] Broemeling [10]
		• Body Mass Index		
		• Alcohol use		
		• Blood pressure		

***Treatment goals and outcomes***				
Safety	Minimizing or eliminating inappropriate prescribing improves quality of care and health outcomes.	Safety - Risk management plan and use of medication alerts	Audit Audit	RACGP [20]
Diabetes mellitus	The percentage of patients with diabetes mellitus for whom the ideal treatment goal of HbA1c* < 7% is met.	Management of diabetes mellitus (HbA1c readings)	Audit	CIHI [18] NHHRC [17]

* HbA1c is a test that measures the amount of glycosylated haemoglobin in the blood - levels below 6% are normal; a person with diabetes mellitus should aim to keep their levels below 7% to reduce the risk of diabetic complications.

To evaluate the impact of the EPHS on health service utilisation, community viability and satisfaction in Elmore and its hinterland, sentinel indicators include: community participation within health service planning; community experience of and satisfaction with health services; indicators of population composition and growth, employment trends, and health service multiplier effects in the local economy[24].

### Data sources

The quantitative data for indicators of health service performance, sustainability and quality are being obtained by an annual comprehensive health service record audit and biennial community surveys. These quantitative data will be analysed using descriptive statistics that monitor trends over the period of the study.

The comprehensive health service audit is examining medical record data, billing and financial data, human resources records, registry data (e.g. Australian Childhood Immunisation Register and the Victorian Cervical Cytology Register) and government reports to the practice (e.g. Practice Incentive Payments and Service Incentive Payments). All data are being extracted by an EPHS practice nurse and provided in a de-identified and aggregated format to the external university research team to ensure utmost privacy and confidentiality of patient and health service records.

Further quantitative data are to be obtained through a biennial community survey that is to be delivered to all residents in the catchment area and is designed to obtain information in relation to health service utilisation, satisfaction and need, in addition to health risk behaviours.

The qualitative data that explores sustainability of the service and the impact of the EPHS on the local community are being obtained by in-depth interviews with key stakeholders and document analysis. These data will be transcribed and thematically analysed and triangulated with the quantitative data for validation and clarification of the data. As new understandings emerge through this iterative and reflective process, new indicators will be developed or existing indicators may be modified.

### Knowledge translation

Engagement with the local community is essential for this study as the current health service grew from community activism in collaboration with the local doctor and regional community health service. A detailed communication strategy, including community forums and articles in the local newspaper, will ensure that all stakeholders and community residents are kept informed of the purpose and conduct of the evaluation study. The conduct of the research and dissemination of findings will be guided by a Reference Group that will be comprised of representatives from the local community (leaders, businesses and community groups), regional health authorities, and general practice support agencies (e.g. Divisions of General Practice and general practice education and training services). The research team will provide information to the community through plain language summaries, oral presentations and presence at key community events.

### Ethics

The study has obtained ethics approvals from Monash University Human Research Ethics Committee (CF08/0419- 2008000176; CF08/0238 - 2008000089; CF08/2434 - 2008001256; CF10/2540 - 20100001423).

## Discussion

Ongoing monitoring and evaluation of successful rural health service models is needed to provide sound empirical evidence of what makes a primary health care service sustainable and effective in a rapidly changing health policy environment in order to provide an evidence base that can inform health service policy makers and other rural health services.

This iterative evaluation framework and methodology aims to ensure the ongoing monitoring of service activity and health outcomes associated with the service that allows researchers, health service providers and administrators to assess the extent to which health service objectives have been met; the factors that have helped or hindered achievements; what worked or did not work well and why; what aspects of the service could be improved and how; what benefits have been realised and for whom; the level of community satisfaction with the service; and the impact of a health service on community viability.

This evaluation focuses on health service performance and capacity rather than focussing on how well a particular disease is treated. This raises challenges in identifying appropriate indicators as primary health care organisation and function is complex, dynamic and composed of both measurable and unmeasurable elements[23] that are subject to many external influences that can impede performance, sustainability and quality of care. The scope and breadth of ideal indicators for the components of each domain in the research literature is more extensive than those selected for this study. The selection of indicators was limited principally by the types of data items that are stored in paper records and electronic databases that are reliable, valid and readily extracted.

This study also provides an opportunity to assess, over time, the effect of locally available health promotion, preventive and early intervention programs on health literacy and health status of a small rural community. As it is possible to relate the EPHS catchment to a geographically circumscribed hinterland, this evaluation may examine the associations between sustainable local health services and community viability and prosperity through examining community satisfaction, growth, employment, and multiplier effect indicators. Hence, building on previous studies that highlighted the significance of rural hospitals and health services to the local economy and fortune of rural communities [25], the findings from this study may assist health authorities and other small rural communities to benchmark what services are appropriate and successful.

The danger in not undertaking such comprehensive longitudinal research to identify "what works well, where and why" is the risk of widening health differential between urban and rural communities; increasing costs of health care treatment to individuals and to government; and reduced implementation of best practice care equitably to all communities.

The need to provide equitable access to quality primary health services for Australia's rural communities is essential and urgent. However, the evidence base for rural health service design remains sparse. This longitudinal evaluation will contribute significant knowledge to how a primary health service can be evaluated and play a role in reducing the Australian rural-metropolitan health disparity.

## Competing interests

The authors declare that they have no competing interests.

## Authors' contributions

RT, JSH and LK conceived and developed the design of the study, obtained the ethics applications and drafted the manuscript. PB obtained the ethics application and contributed to drafting and revising the manuscript. AA and KT contributed to the conception and design of the study and revising the manuscript. All authors read and approved the final manuscript.

## Pre-publication history

The pre-publication history for this paper can be accessed here:

http://www.biomedcentral.com/1472-6963/11/52/prepub
